# Legal Services for Veterans (LSV): Protocol for evaluating the grant-based LSV initiative supporting community organizations’ delivery of legal services to veterans

**DOI:** 10.1371/journal.pone.0297424

**Published:** 2024-04-16

**Authors:** Bo Kim, Beth Ann Petrakis, Ida Griesemer, Samantha K. Sliwinski, Amanda M. Midboe, Rebecca A. Raciborski, Thomas H. Byrne, Madolyn B. Gingell, Jessica Blue-Howells, Sean C. Clark, Jack Tsai, Kim L. L. Harvey, D. Keith McInnes

**Affiliations:** 1 Center for Healthcare Organization and Implementation Research, VA Boston Healthcare System, Boston, Massachusetts, United States of America; 2 Harvard Medical School, Boston, Massachusetts, United States of America; 3 Center for Healthcare Organization and Implementation Research, VA Bedford Healthcare System, Bedford, Massachusetts, United States of America; 4 Center for Innovation to Implementation, VA Palo Alto Health Care System, Menlo Park, California, United States of America; 5 University of California at Davis School of Medicine, Sacramento, California, United States of America; 6 Center for Mental Healthcare and Outcomes Research, Central Arkansas Veterans Healthcare System, North Little Rock, Arkansas, United States of America; 7 Behavioral Health Quality Enhancement Research Initiative, Central Arkansas Veterans Healthcare System, North Little Rock, Arkansas, United States of America; 8 Boston University School of Social Work, Boston, Massachusetts, United States of America; 9 Veterans Health Administration Homeless Programs Office, Washington, District of Columbia, United States of America; 10 University of Texas Health Science Center at Houston, Houston, Texas, United States of America; 11 Boston University School of Public Health, Boston, Massachusetts, United States of America; Public Library of Science, UNITED STATES

## Abstract

**Background:**

1.8 million Veterans are estimated to need legal services, such as for housing eviction prevention, discharge upgrades, and state and federal Veterans benefits. While having one’s legal needs met is known to improve one’s health and its social determinants, many Veterans’ legal needs remain unmet. Public Law 116–315 enacted in 2021 authorizes VA to fund legal services for Veterans (LSV) by awarding grants to legal service providers including nonprofit organizations and law schools’ legal assistance programs. This congressionally mandated LSV initiative will award grants to about 75 competitively selected entities providing legal services. This paper describes the protocol for evaluating the initiative. The evaluation will fulfill congressional reporting requirements, and inform continued implementation and sustainment of LSV over time.

**Methods:**

Our protocol calls for a prospective, mixed-methods observational study with a repeated measures design, aligning to the Reach Effectiveness Adoption Implementation Maintenance (RE-AIM) and Integrated Promoting Action on Research Implementation in Health Services (i-PARIHS) frameworks. In 2023, competitively selected legal services-providing organizations will be awarded grants to implement LSV. The primary outcome will be the number of Veterans served by LSV in the 12 months after the awarding of the grant. The evaluation has three Aims. Aim 1 will focus on measuring primary and secondary LSV implementation outcomes aligned to RE-AIM. Aim 2 will apply the mixed quantitative-qualitative Matrixed Multiple Case Study method to identify patterns in implementation barriers, enablers, and other i-PARIHS-aligned factors that relate to observed outcomes. Aim 3 involves a mixed-methods economic evaluation to understand the costs and benefits of LSV implementation.

**Discussion:**

The LSV initiative is a new program that VA is implementing to help Veterans who need legal assistance. To optimize ongoing and future implementation of this program, it is important to rigorously evaluate LSV’s outcomes, barriers and enablers, and costs and benefits. We have outlined the protocol for such an evaluation, which will lead to recommending strategies and resource allocation for VA’s LSV implementation.

## Introduction

Over 90% of low-income Americans do not receive adequate legal assistance for their civil legal problems, according to the Legal Services Corporation, which provides legal assistance to low-income Americans [1]. It is also known that as much as 10% of the nation’s 18 million Veterans need legal services [2]. Issues such as eviction proceedings or accessing disability benefits often cannot be adequately addressed without advice or support from a legal professional. Low-income Veterans who cannot afford to pay for legal services are at a particular disadvantage [3]. For Veterans experiencing or at risk for homelessness, access to free legal services that support social determinants of health (e.g., safe housing) has been associated with reduced symptoms of anxiety and posttraumatic stress disorder and improved overall health [4].

While VA, by regulation, cannot directly provide legal services [4], it actively connects Veterans to community legal assistance resources. Some VA facilities host free legal clinics for Veterans operated by non-VA legal specialists. These arrangements enable interactive care collaborations between clinicians and legal service providers, working in the same location and delivering holistic care for Veterans. Also, through its Supportive Services to Veteran Families program, VA funds community-based nonprofits to provide services to Veterans, and many of these nonprofits subcontract with legal service providers to support Veterans [5]. However, many legal needs of Veterans remain unmet. Veterans not yet connected with VA, those residing in rural areas [6, 7], or those requiring more intensive and longer-duration legal assistance [8] are especially at risk for unmet need.

Enacted in 2021, Public Law 116–315 (focused on Veterans experiencing or at risk for homelessness) authorizes VA to award grants to organizations to provide legal services to Veterans (e.g., in the areas of housing, family law, income support, criminal defense, and discharge upgrades) [9]. These organizations may be Veterans Service Organizations, law schools’ legal clinics, legal services organizations, bar associations, or other nonprofits or entities that VA deems appropriate. At least 10% of the funding must serve eligible women Veterans. The law also emphasizes serving Veterans in rural and tribal communities.

Veterans Justice Programs (VJP), under VA’s national Homeless Programs Office, manages this grant-based Legal Services for Veterans (LSV) initiative. VJP’s core functions are conducting outreach to justice-involved Veterans and facilitating their access to VA services. VJP plays an active role in facilitating Veterans’ access to legal services, including the above-mentioned VA-hosted pro-bono legal clinics. The LSV program will allow broad expansion of legal services to Veterans with legal needs. To oversee LSV operations, VJP has added new staff positions and an administrative infrastructure that utilizes an electronic Grants Management System to coordinate communication with and compliance monitoring across the grantee organizations. VJP’s grant management tasks will also include raising awareness among potential applicant organizations about this funding opportunity and providing technical assistance to grantee organizations for getting LSV operations underway.

In the first year of the initiative, approximately 75 competitively selected legal services-providing organizations external to VA will be awarded one-year grants of up to $150,000 each. This amount is meant to cover annual salary and benefit costs for approximately one attorney position, as well as data collection, reporting, and other administrative costs associated with the grant. Grantee organizations will be public or nonprofit private entities with adequate capacity and resources to administer a grant and deliver legal services. VJP is structuring LSV informational and application materials to encourage grantee organizations to follow established models of legal services delivery that take health care services into account, such as the Medical-Legal Partnership model [4, 10, 11]. The grantee organizations, especially given their varying types from Veterans Service Organizations to bar associations, will still have the flexibility to devise their own operationalizations of LSV. This will likely result in diverse processes of legal service provision under LSV, warranting careful examination and leading to an opportunity to assess different structures and practices for LSV.

Accordingly, a formal evaluation is imperative to understand diverse LSV processes and their resulting impact. To inform sustainment, scale-up, and spread of LSV beyond its initial implementation period, the evaluation must not only consider the outcomes of LSV implementation but also *how* and *why* certain outcomes were reached or not reached, which may help elucidate what influences the delivery of legal services to Veterans. VA must report to Congress the impact of LSV, for which an economic evaluation of LSV implementation will also be necessary.

In 2022, VJP, the VA National Center on Homelessness among Veterans (which leads evaluation and research initiatives on topics of homelessness) and implementation evaluation experts drawn from research centers funded by VA Health Services Research & Development co-developed a plan to formally evaluate LSV implementation. After a competitive peer-review process, a three-year grant was awarded by the VA Quality Enhancement Research Initiative for this partnered LSV evaluation, collaboratively funded by the Homeless Programs Office. This article describes the LSV evaluation protocol. The LSV evaluation findings will be published in subsequent manuscripts.

## Materials and methods

### Overview

We will conduct the evaluation of LSV implementation as a prospective, mixed-methods observational study with a repeated measures design. In 2023, competitively selected legal services-providing organizations will be awarded a one-year grant to implement LSV. The primary outcome will be the number of Veterans served by LSV in the 12 months after the awarding of the grant. We will align our evaluation to two frameworks, Reach Effectiveness Adoption Implementation Maintenance (RE-AIM) [12] and Integrated Promoting Action on Research Implementation in Health Services (i-PARIHS) [13]. We will first use RE-AIM to comprehensively assess LSV implementation outcomes (Aim 1). Those outcomes will then inform the purposive selection of grantee organizations at which to use i-PARIHS to delineate both common and heterogeneous contextual factors that influence how LSV is implemented (Aim 2). Finally, we will conduct a mixed-methods economic evaluation [14] of LSV using data from Aims 1 and 2 (Aim 3). Below, we describe in detail the planned evaluation procedures for each Aim. S1 File provides the Strengthening the Reporting of Observational Studies in Epidemiology (STROBE) guidelines [15] that we have consulted and will continue to consult in reporting our work. This project was determined to be non-research by the VA Boston Research and Development Service, and therefore does not require oversight by the Institutional Review Board (IRB).

### Aim 1: Assess the outcomes of LSV implementation

#### Aim 1 overview

First, leveraging regularly collected data from VJP’s Grants Management System of audits and compliance monitoring of LSV, we will assess, per RE-AIM: (i) The number of Veterans served by LSV and their proportions for key populations specified in the law (e.g., women) (*Reach*), (ii) grantee organizations’ representativeness of target regions and communities (*Adoption*), (iii) the proportion of grantee organizations implementing their intended LSV services (*Implementation*), and (iv) the extent to which grantee organizations sustain LSV over time (*Maintenance*). Then, aligning to RE-AIM’s *Effectiveness* dimension, we will administer a voluntary feedback questionnaire to Veterans receiving LSV services, with an optional follow-up interview, to assess the extent to which Veterans perceive their legal needs to be met by LSV and are satisfied.

#### Aim 1 data collection

Data to be collected for Aim 1, along with their alignment to RE-AIM, are outlined in Table 1.

**Table 1:** Assessments to be conducted for Aim 1.

Data source	Assessment (RE-AIM^a^ outcomes)	Target(s)	Timepoint(s)
**Grants Management System**	Reach: Number of Veterans served by LSV to date, proportion of them who are women, and proportion of them who are experiencing or at risk for homelessness	Grantee organizations LSV initiative overall	6, 12, and 18^b^ months after the awarding of the grant
	Adoption: Proportion of states to date that have grantee organizations among all states from which qualifying grant applications were received, as well as proportions of grantee organizations that serve rural and tribal communities	LSV initiative overall	
	Implementation: Whether delivering none, some, or all of the legal services that were proposed in the grant application	Grantee organizations	
	Implementation: Proportions of grantee organizations that are delivering none, some, or all of the legal services that were proposed in the grant application	LSV initiative overall	
	Maintenance: Reassessment of the *Reach*, *Adoption*, and *Implementation* measures described above	Grantee organizations LSV initiative overall	24^b^ months after the awarding of the grant
**Veteran feedback questionnaire and follow-up interview**	Effectiveness: Extent to which Veterans served by LSV perceive their legal needs were met and are satisfied with the legal services received	LSV initiative overall (Veterans who are receiving / received one or more legal services from a grantee organization)	6 months after start of service(s)

^a^Reach Effectiveness Adoption Implementation Maintenance

^b^For grantee organizations that continue to receive a second year of LSV funding

*Grants Management System data collection*. Through the Grants Management System managed by VJP, grantee organizations will be required to report on a quarterly basis their LSV activity–e.g., the number of Veterans (e.g., overall, women, and experiencing or at risk for homelessness) they served, the types of legal services provided (e.g., housing-, family-, income-, criminal defense-, or discharge upgrade-related), description of legal matters addressed (e.g., eviction notice, child support/custody), whether they provided limited or full representation, and whether the cases they were representing were resolved. In addition to these RE-AIM-aligned data, we will also collect data through the Grants Management System on each organization’s geographic location and whether the organization is (i) a Veterans Service Organization or other public/nonprofit organization, (ii) a legal assistance clinic of a law school, (iii) a legal services organization, (iv) a bar association, or (v) a different type of organization. We will also collect additional information on each organization’s setting, including rurality, homelessness statistics, justice involvement statistics, and size of the Veteran population [16–19].

*Veteran feedback questionnaire data collection*. For the Veteran feedback questionnaire assessing the extent to which Veterans perceive their legal needs to be met by LSV and are satisfied, participants will be recruited as follows: When a Veteran begins receiving services at a grantee organization, the organization will ask the Veteran whether their contact information can be shared with our evaluation team (explaining that the team is helping VA, as the funder of LSV, to learn and improve how LSV supports Veterans). If the Veteran agrees, then the evaluation team will receive their contact information from the grantee organization. After approximately 6 months, we will reach out to the Veteran (checking with the grantee organization if updated contact information is needed) to provide them with information about our evaluation and ask whether they would be willing to complete a questionnaire, and if so, which modality they prefer (e.g., online, via phone). We will obtain informed consent from the Veteran prior to their responding to the questionnaire, either electronically or verbally based on whether they choose to complete the questionnaire online or via phone, respectively. The questionnaire will be entirely voluntary and confidential (not anonymous, to enable the evaluation team to contact them as described above and to offer compensation for their participation as described below), and they will be assured that (i) their responses will be combined with those from other participants and never be reported in a way that makes it possible to identify them individually, (ii) deciding not to complete the questionnaire will have no effect on the legal services that they receive, and (iii) the grantee organization will not be notified of whether they choose to complete the questionnaire.

We designed a brief (< 10 minutes) questionnaire, learning from the Legal Services Corporation’s examples of gathering feedback from clients [20]. The questionnaire asks the Veteran respondent to identify the matters for which they received legal services from the grantee organization (e.g., housing, family, income, criminal defense, discharge upgrade) and also the type of either limited (e.g., legal advice only) or extended (e.g., negotiated settlement with or without litigation) services that they received. The questionnaire asks to identify the location at which the respondent is/was receiving legal services, and it also asks about their case’s outcome (with a “not applicable” option if their case is not yet resolved). The questionnaire asks the Veteran respondent’s satisfaction with the extent to which LSV helped meet their legal needs (five-point Likert scale from Very Satisfied to Very Unsatisfied). An open-ended question asks for additional comments and/or suggestions, and also included are questions regarding their age, gender, and race/ethnicity. The questionnaire concludes by asking whether they would be interested in participating in a follow-up interview to further share their LSV experiences. Veteran respondents will be offered $10 as compensation for their participation. The follow-up interview with interested participants will focus on collecting data regarding the context under which they have responded to the questionnaire, specifically asking them to elaborate on experiences that led to their questionnaire responses.

#### Aim 1 data analysis

*Grants Management System data analysis*. By analyzing Grants Management System data across the grantee organizations, we will assess their *Reach* and *Implementation* at 6, 12, and 18 months after the awarding of the grant (data after 12 months will be available if grants are renewed and for grantee organizations that continue to receive a second year of LSV funding), and examine these measures’ variation across organizations and across different timepoints. We will examine the differences in the measurements’ mean change across different characteristics of the grantee organizations and of the grantee organizations’ settings. Similarly, for *Maintenance*, we will assess the measurements’ mean change from 18 to 24 months, then examine the differences in the means across different characteristics of the grantee organizations and of the grantee organizations’ settings. Differences in means will be examined using repeated measures analyses of variance, with the alpha for statistical significance adjusted for multiple comparisons. For the LSV initiative overall, we will assess *Reach*, *Adoption*, and *Implementation* at 6, 12, and 18 months, then again at 24 months to examine *Maintenance*.

LSV’s goal is to deliver legal services to approximately 12,000 Veterans in its first year. Across the 75 grantee organizations to be awarded the funding, we will initially regard organization-specific target *Reach* to be a weighted proportion of 12,000, relative to the size of their local Veteran population [16]–i.e., a grantee organization with a larger local Veteran population will have a higher proportion of 12,000 as their target *Reach*. We will then further refine the target as we track *Reach* over time through the Grants Management System. For measuring *Implementation*, we recognize that (i) available legal services expertise and resources will likely vary widely across grantee organizations and (ii) an organization may be meeting Veterans’ needs (and thus delivering LSV as intended) whether they deliver many or few types of legal services. We will therefore categorize *Implementation* as high, medium, or low, by whether a grantee organization delivers none, some, or all of the legal services (e.g., family law, access to health care, employment law) that it proposed to deliver in its grant application.

*Veteran feedback questionnaire data analysis*. For analyzing *Effectiveness* using Veteran feedback data, we will take a simultaneous complementary mixed quantitative-qualitative approach [21], concurrently collecting and analyzing quantitative and qualitative data, then using them complementarily to provide breadth and depth of understanding, respectively. Specifically, we will compute frequencies and proportions of the closed-ended questionnaire responses and explore variation by age, gender, and race/ethnicity. We will conduct chi-square analyses to examine whether there are statistical associations between responses to different questions. We will examine whether respondent age, gender, and race/ethnicity are associated with the type of legal services received (e.g., family law, access to health care, employment law), and what types of legal services are most commonly received (e.g., related to VA benefits, housing, family issues, or consumer issues) [4]. We will stratify satisfaction findings by case outcomes, and we will also examine differences in satisfaction and in characteristics between respondents who are interested versus not interested in participating in the optional follow-up interview.

For open-ended questionnaire responses and interview data, we will use a framework-guided rapid analysis approach [22] aligned to the questionnaire items. We will use an item-based structured template to summarize transcripts, then consolidate the summaries into matrices by item. Four evaluation team members will create the summaries, each serving as the primary summarizer for one-fourth of the data and as the secondary reviewer for another one-fourth. Discrepancies will be resolved through consensus discussions [22]. For each questionnaire item, key themes based on the summaries and their associated examples will be consolidated into a report. Specifically, two of the four members will identify key themes for each item, and each will review and discuss the other’s work. The third and fourth members will review revised summaries for all items and all four members will meet to discuss and finalize them. Mixed-methods interpretation of findings will account for biases and variations in participant characteristics.

### Aim 2: Identify the barriers to and enablers of LSV implementation

#### Aim 2 overview

Data will be collected through semi-structured interviews at 15 grantee organizations with legal specialists and LSV leads who oversee their organization’s operationalization of the LSV program. The organizations will be purposively selected to vary by type, geographic location, rurality, and RE-AIM measures examined under Aim 1 (sequential sampling mixed methods). To inform future sustainment, scale-up, and spread of LSV, we will conduct a mixed-methods Matrixed Multiple Case Study (MMCS) [23], aligning to the four i-PARIHS constructs to delineate relevant characteristics of LSV operations (*Innovation*), grantee organizations (*Recipients*), local-to-national settings (*Context*), and implementation strategies (*Facilitation*). We will employ sequential explanatory mixed methods by using these characteristics to further contextualize Aim 1 findings.

#### Aim 2 data collection

To purposively select the 15 grantee organizations at which to conduct semi-structured interviews with legal specialists and LSV leads, we will take a sequential sampling mixed methods approach. We will prioritize varying the grantee organizations by

- *Reach* (high, medium, low; trichotomizing [24] organizations by those who belong in the upper, middle, and lower third of *Reach* at 6 months after the awarding of the grant)
- Organization type (e.g., Veterans Service Organizations, law schools’ legal clinics, legal services organizations, bar associations, or other nonprofits or entities that VA deems appropriate)
- Geographic representation
- Urban-rural designation based on the Rural-Urban Continuum Codes [19]

At each of the selected grantee organizations, we will interview a legal specialist and an LSV lead at two timepoints, the first at 6 months after the organizations are awarded the grant and the second as a follow-up after the end of the organizations’ grant period. We will contact each organization’s director for help in identifying staff who are potential participants; the director will not be involved otherwise, unless the director is a legal specialist or an LSV lead. Potential participants will be assured that (i) participation is voluntary and confidential (not anonymous, to enable the evaluation team to contact them as described above), (ii) their decision to participate will not be shared with the director, and (iii) the information that they choose to share during the interview will be combined with those from other participants and never be reported in a way that makes it possible to identify them individually. Verbal informed consent will be obtained from all participants.

Interviews will be phone-based or conducted via a virtual communication software and take approximately 45 minutes. Interview questions will be about types of legal services provided and legal matters addressed by LSV. Especially for participants from grantee organizations for which Aim 1 *Implementation* data show that they are not delivering some of their originally proposed LSV services (e.g., housing-, family-, income-, criminal defense-, and discharge upgrade-related legal services), we will ask about perceived reasons. We will also ask about characteristics of LSV operations, grantee organizations (e.g., experience with other grant funding, history of providing legal aid or public-interest law services), local-to-national settings, and implementation strategies that they deem relevant to their LSV implementation and sustainment. Interviews will be audio-recorded and transcribed verbatim. If a participant prefers to not be recorded, detailed notes will be taken by a second evaluation team member.

#### Aim 2 data analysis

Assessments to be conducted for Aim 2, along with their alignment to i-PARIHS, are outlined in Table 2. We will use a framework-guided rapid analysis approach similar to that described above for Aim 1 qualitative analysis, except now aligned to i-PARIHS constructs *Innovation*, *Recipients*, *Context*, and *Facilitation*. Because participating grantee organizations may have changed in their *Reach* and *Implementation* levels between the two interview timepoints, when summarizing interviews conducted at the second timepoint, we will note how findings have changed between the two timepoints and whether the changes may relate to an organization’s move to a different level of *Reach* and/or *Implementation* since the first timepoint.

**Table 2:** Assessments to be conducted for Aim 2.

Data source	Assessment (i-PARIHS^a^ constructs)
**Interviews with legal specialists and LSV leads at grantee organizations**	Innovation: Relevant characteristics of LSV operations (e.g., whether what LSV entails is clear to the grantee organization, whether LSV fits with the grantee organization’s existing practices)
	Recipients: Relevant characteristics of grantee organizations (e.g., whether LSV requires unique skills of the grantee organization’s employees, whether there are existing networks to leverage)
	Context: Relative characteristics of local-to-national settings (e.g., whether there is leadership support for LSV, whether there are other incentives/mandates that affect LSV implementation)
	Facilitation: Relevant characteristics of implementation strategies (e.g., support from VA for implementing LSV, other tools that enable better clarification/assessment of implementation)
**Findings from Aims 1 and 2 for mixed-methods MMCS** ^b^	Cross-organization trends in influencing factors associated with varied extents (high, middle, or low) of LSV implementation based on *Reach* and *Implementation*

^a^i-PARIHS: Integrated Promoting Action on Research Implementation in Health Services

^b^MMCS: Matrixed Multiple Case Study

We will also assess the extent to which grantee organizations are following established models of legal services delivery that take health care services into account. For this assessment, in addition to using i-PARIHS to structure the template for summarizing transcripts, we will build sections into the template for noting whether and how grantee organizations perform key Medical-Legal Partnership activities [25] and the extent to which such activities align to core Medical-Legal Partnership elements [26]. Key Medical-Legal Partnership activities include “providing legal assistance in the health care setting, educating health professionals about the significance of social determinants of health, and working toward policy change by addressing laws standing in the way of good health” [27]. Since the evidence base for Medical-Legal Partnerships is still being established and LSV implementation currently does not require fulfillment of Medical-Legal Partnership activities, we will approach this mainly as an exploration of whether observed alignments of grantee LSV work to Medical-Legal Partnership activities trend with LSV outcomes, for the purposes of informing ongoing and future LSV implementation. Such exploration is especially timely, given that LSV is a new initiative that is looking to build its evidence base and be further refined to maximize its effectiveness.

We will employ sequential explanatory mixed methods as part of MMCS, using qualitatively analyzed data from interviews to contextualize quantitative data on LSV implementation outcomes. We will use the summaries of the i-PARIHS constructs to identify factors influencing LSV implementation per organization, reaching consensus on each factor as having (i) been present, somewhat present, or minimally present and (ii) had an enabling, neutral, hindering, or unclear effect on LSV implementation. Consensus on these designations will be sought in two steps. First, two or more evaluation team members will independently review the data to each propose initial designations and their interpretations, accounting for any variations in the data’s availability and completeness. Next, consensus-reaching discussions will be held to finalize the designations and interpretations. We will curate the examined data into a sortable matrix. Using the matrix, we will assess the data for cross-organization trends in factors that are associated with high, middle, and low *Reach* and *Implementation* levels as defined above. Analysis will be led by four team members and reviewed by all team members.

### Aim 3: Examine the costs and benefits of LSV implementation

#### Aim 3 overview

We will apply Dopp and colleagues’ simultaneous complementary mixed-methods economic evaluation approach [14]. We will quantitatively assess VA’s monetary costs to achieve the outcomes measured under Aim 1 and describe variations in grantee organizations’ outcomes as a function of cost and contextual characteristics. We will qualitatively assess the perceived costs and benefits of LSV using the interviews described under Aim 2. Findings will identify indicators for use in future cost-effectiveness analysis of subsequent LSV evaluations and generate hypotheses about contextual characteristics linked to greater cost-effectiveness.

#### Aim 3 data collection

Aim 3 will use LSV implementation outcomes measured under Aim 1 and data collected through the interviews described under Aim 2. To enable mixed-methods economic evaluation, increasingly used in assessing implementation endeavors [14, 28], we will qualitatively examine the interview data on LSV’s perceived costs and benefits [29]. Site selection and participant-facing procedures will be as described under Aim 2. For data collection, the semi-structured interview guides will include questions about participants’ perceptions on three topics–(i) costs (both time and resources) and benefits (from Veteran- to society-level benefits) of LSV implementation, (ii) costs and benefits of sustaining LSV over time, and (iii) expected impact if LSV was not available. We will additionally ask what participants consider to be Veteran-, organizational-, and/or society-level indicators of LSV implementation and sustainment. Drawing on rapid-cycle approaches to incorporating collaborators’ perspectives in cost analyses [30], we will also ask participants who they perceive as key entities vis-à-vis cost when LSV is implemented (e.g., Veterans’ caregivers, dependents, etc.).

#### Aim 3 data analysis

Table 3 outlines analyses to be conducted for Aim 3. Cost analyses for decisions about implementation and sustainment are most informative for policy when performed from the perspective of the funding organization. Because the Homeless Programs Office is VA’s primary entity that is tasked with implementing LSV, and because this evaluation is meant to assist the Homeless Programs Office directly in optimizing LSV implementation, the economic evaluation is conducted from this perspective. The total cost of LSV implementation comprises the sum of the awards to grantee organizations and administrative costs. Administrative costs include adding staff positions to VJP under the Homeless Programs Office to manage the LSV initiative by using the Grants and Payment Management Systems, raising awareness about the grant opportunity among potential grantees, guiding potential grantees through the application process, and conducting regular compliance monitoring/audits of grantee organizations. We will examine the cost per Veteran served through LSV for the LSV initiative overall and by grantee organizational characteristics. Number of Veterans served will be based on *Reach* data collected under Aim 1. To examine potential relationships between contextual factors that may affect implementation cost, we will also analyze whether and how characteristics of the grantee organizations and their settings (as described for Aim 1) are associated with *Reach*.

**Table 3:** Assessments to be conducted for Aim 3.

Data source	Assessment	Target(s)
**Grants Management System**	Impact of the grant budget: Cost per Veteran served by LSV	Grantee organizations LSV initiative overall
**Interviews with legal specialists and LSV leads at grantee organizations**	Perceived costs and benefits of LSV	Purposively sampled 15 grantee organizations

We will conduct rapid qualitative analysis [22] (as described under Aim 2) of data collected using the cost- and benefit-related interview questions. We will compare findings across the two interview timepoints to assess whether the first timepoint’s findings differ by grantee organizations exhibiting high, middle, or low *Reach* and *Implementation* levels, and whether the second timepoint’s findings differ by organizations that stayed in or changed from their levels of *Reach* and/or *Implementation* at the first timepoint. Taking the simultaneous complementary mixed-methods economic evaluation approach [14], we will examine whether the quantitative variation seen in cost-per-Veteran data from the participating grantee organizations are related to differences in perceived costs and benefits that are qualitatively identified. Our analyses will benefit from quantitative data providing breadth of understanding and qualitative data providing depth of understanding [14].

## Discussion

The LSV initiative breaks new ground for VA through grant-based funding of community organizations that provide legal services to Veterans. The protocol described here indicates the methods for evaluating LSV implementation–an implementation that involves varying types of grantee organizations and processes for operationalization. To understand these processes and their varied impact, this evaluation aligns with conceptual frameworks and applies novel mixed methods to (i) assess the outcomes of, (ii) identify the barriers to and enablers of, and (iii) examine the costs and benefits of LSV implementation.

In the short term, achieving the evaluation Aims outlined above will deliver information for VJP to optimize LSV implementation by maximizing benefits and minimizing costs. Actionable information will include ways for VJP to better support grantee organization operations, as well as population- and/or organization-specific adaptations to better meet Veterans’ needs. In the longer term, findings will contribute to strengthening future LSV evaluations, including determining potential quantitative *Effectiveness* measures that can both be feasibly gathered and align to perceived indicators of LSV success identified through this current evaluation. Findings will also shape additional considerations for future LSV evaluations, such as whether and how other VA- and/or community-based services are impacted by LSV’s success, and importantly, they will inform similar initiatives both within and outside VA to support the health and well-being of individuals through enhancing their access to legal services.

### Limitations and anticipated challenges

As a real-world program implementation in diverse settings and amidst myriad contextual variations, we expect LSV to have heterogeneous outcomes across grantee organizations and across time. This evaluation examines a novel program of a federally-funded healthcare system that awards grants to legal service providers to serve patients. Relying on measures of central tendency (e.g., cross-organization averages for the RE-AIM-aligned implementation outcomes under Aim 1) to examine whether LSV works may lead to a considerable missed opportunity to examine how and why a program works or does not work under varied circumstances. We will attempt to address this limitation in Aim 2 by subjecting our collected data to MMCS, an approach specifically designed to capitalize on heterogeneities among participating sites. Use of MMCS may also provide a countermeasure to the possibility that the data lack assumptions for reliable analyses of variance.

Moreover, when analyzing data from the interviews with legal specialists and LSV leads at the 15 purposively selected grantee organizations, we will keep in mind that purposive sampling is meant for illustrative inferences about what is possible. This is unlike probability sampling for quantitative studies, which leads to drawing statistical inferences about specified possibilities’ prevalence [31, 32]. We will thus not characterize findings based on the number of interview participants linked to a finding, noting such counts only to ensure that all data are accounted for [32, 33].

## Conclusions

Many Veterans need legal help for challenges that diminish or impede their physical health, mental health, family relationships, housing stability, and community involvement and integration. The novel LSV initiative aims to help Veterans in need of legal assistance. As a new initiative, LSV’s outcomes, barriers and enablers, and costs and benefits are unknown. Our planned LSV evaluation outlined in this article has been specifically designed to fill these knowledge gaps. Evaluation findings will culminate in recommended strategies and resource allocation for LSV to target Veterans’ unmet legal needs and thereby decrease their risk for experiencing both health and social challenges.

## Supporting information

S1 File STROBE statement. (PDF)

## References

[1] The Justice Gap: The Unmet Civil Legal Needs of Low-income Americans 2023. https://justicegap.lsc.gov/.

[2] Timko C, Taylor E, Nash A, Blonigen D, Smelson D, Tsai J, et al. National Survey of Legal Clinics Housed by the Department of Veterans Affairs to Inform Partnerships with Health and Community Services. J Health Care Poor Underserved. 2020;31(3):1440–56. doi: 10.1353/hpu.2020.0104 33416704 PMC8215811

[3] Tsai J, Jenkins D, Lawton E. Civil Legal Services and Medical-Legal Partnerships Needed by the Homeless Population: A National Survey. Am J Public Health. 2017;107(3):398–401. doi: 10.2105/AJPH.2016.303596 28103065 PMC5296685

[4] Tsai J, Middleton M, Villegas J, Johnson C, Retkin R, Seidman A, et al. Medical-Legal Partnerships At Veterans Affairs Medical Centers Improved Housing And Psychosocial Outcomes For Vets. Health Aff (Millwood). 2017;36(12):2195–203. doi: 10.1377/hlthaff.2017.0759 29200329

[5] SSVF Legal Services Frequently Asked Questions 2023. https://www.va.gov/HOMELESS/ssvf/docs/Legal_Services_FAQ.pdf.

[6] Statz M, Termuhlen P. Rural Legal Deserts Are a Critical Health Determinant. Am J Public Health. 2020;110(10):1519–22. doi: 10.2105/AJPH.2020.305807 32816549 PMC7483108

[7] Speldewinde CA, Parsons I. Medical-legal partnerships: the role of mental health providers and legal authorities in the development of a coordinated approach to supporting mental health clients’ legal needs in regional and rural settings. Rural Remote Health. 2015;15(4):3387. 26556553

[8] Timko C, Tsai J, Taylor E, Smelson D, Blonigen D, Nash A, et al. Clients of VA-Housed Legal Clinics: Legal and Psychosocial Needs When Seeking Services and Two Months Later. J Veterans Stud. 2020;6(1):239–49. doi: 10.21061/jvs.v6i1.167 34466762 PMC8404205

[9] Public Law 116–315 Section 4202 2022. https://www.congress.gov/116/plaws/publ315/PLAW-116publ315.pdf.

[10] Beardon S, Woodhead C, Cooper S, Ingram E, Genn H, Raine R. International Evidence on the Impact of Health-Justice Partnerships: A Systematic Scoping Review. Public Health Rev. 2021;42:1603976. doi: 10.3389/phrs.2021.1603976 34168897 PMC8113986

[11] Partnerships across the U.S. 2022. https://medical-legalpartnership.org/partnerships.

[12] Glasgow RE, Vogt TM, Boles SM. Evaluating the public health impact of health promotion interventions: the RE-AIM framework. Am J Public Health. 1999;89(9):1322–7. doi: 10.2105/ajph.89.9.1322 10474547 PMC1508772

[13] Harvey G, Kitson A. PARIHS revisited: from heuristic to integrated framework for the successful implementation of knowledge into practice. Implement Sci. 2016;11:33. doi: 10.1186/s13012-016-0398-2 27013464 PMC4807546

[14] Dopp AR, Mundey P, Beasley LO, Silovsky JF, Eisenberg D. Mixed-method approaches to strengthen economic evaluations in implementation research. Implement Sci. 2019;14(1):2. doi: 10.1186/s13012-018-0850-6 30635001 PMC6329154

[15] von Elm E, Altman DG, Egger M, Pocock SJ, Gøtzsche PC, Vandenbroucke JP. Strengthening the Reporting of Observational Studies in Epidemiology (STROBE) statement: guidelines for reporting observational studies. Bmj. 2007;335(7624):806–8. doi: 10.1136/bmj.39335.541782.AD 17947786 PMC2034723

[16] National Center for Veterans Analysis and Statistics 2022. https://www.va.gov/vetdata/veteran_population.asp.

[17] National Institute of Corrections 2022. https://nicic.gov/projects/state-statistics-information.

[18] Point-in-Time Counts of Homelessness 2022. https://www.hudexchange.info/resource/3031/pit-and-hic-data-since-2007.

[19] Rural-Urban Continuum Codes 2022. https://www.ers.usda.gov/data-products/rural-urban-continuum-codes.aspx.

[20] Outcomes Case Studies 2022. https://www.lsc.gov/our-impact/civil-legal-outcomes/case-studies.

[21] Palinkas LA, Aarons GA, Horwitz S, Chamberlain P, Hurlburt M, Landsverk J. Mixed method designs in implementation research. Administration and policy in mental health. 2011;38(1):44–53. doi: 10.1007/s10488-010-0314-z 20967495 PMC3025112

[22] Gale RC, Wu J, Erhardt T, Bounthavong M, Reardon CM, Damschroder LJ, et al. Comparison of rapid vs in-depth qualitative analytic methods from a process evaluation of academic detailing in the Veterans Health Administration. Implement Sci. 2019;14(1):11. doi: 10.1186/s13012-019-0853-y 30709368 PMC6359833

[23] Kim B, Sullivan JL, Ritchie MJ, Connolly SL, Drummond KL, Miller CJ, et al. Comparing variations in implementation processes and influences across multiple sites: What works, for whom, and how? Psychiatry Res. 2020;283:112520. doi: 10.1016/j.psychres.2019.112520 31627960

[24] Gelman A, Park DK. Splitting a Predictor at the Upper Quarter or Third and the Lower Quarter or Third. The American Statistician. 2009;63(1):1–8.

[25] Huston RL, Zinn S, Leal-Castanon S. Medical-legal partnerships. Virtual Mentor. 2011;13(8):555–8. doi: 10.1001/virtualmentor.2011.13.8.hlaw1-1108 23137458

[26] Trott J, Lattimore K, Teitelbaum J, Regenstein M. VA Medical-Legal Partnerships: Implementation Guidance and Suggested Measures 2022. https://medical-legalpartnership.org/download/va-mlp-implementation-guidance/.

[27] Beeson T, McAllister BD, Regenstein M. Making the Case for Medical-Legal Partnerships: A Review of the Evidence 2022. https://medical-legalpartnership.org/wp-content/uploads/2014/03/Medical-Legal-Partnership-Literature-Review-February-2013.pdf.

[28] O’Leary MC, Hassmiller Lich K, Frerichs L, Leeman J, Reuland DS, Wheeler SB. Extending analytic methods for economic evaluation in implementation science. Implement Sci. 2022;17(1):27. doi: 10.1186/s13012-022-01192-w 35428260 PMC9013084

[29] Mundey P, Slemaker A, Dopp AR, Beasley LO, Silovsky JF. Qualitative Analysis of Administrator, Provider, and Stakeholder Views on the Costs and Benefits of a Treatment for Problematic Sexual Behavior of Youth. Adm Policy Ment Health. 2020;47(1):126–37. doi: 10.1007/s10488-019-00978-3 31549277

[30] Eisman AB, Quanbeck A, Bounthavong M, Panattoni L, Glasgow RE. Implementation science issues in understanding, collecting, and using cost estimates: a multi-stakeholder perspective. Implement Sci. 2021;16(1):75. doi: 10.1186/s13012-021-01143-x 34344411 PMC8330022

[31] Wood M, Christy R. Sampling for possibilities. Qual Quant. 1999;33:185–202.

[32] Chang Y, Voils CI, Sandelowski M, Hasselblad V, Crandell JL. Transforming verbal counts in reports of qualitative descriptive studies into numbers. West J Nurs Res. 2009;31(7):837–52. doi: 10.1177/0193945909334434 19448052 PMC2784172

[33] Sandelowski M. Real qualitative researchers do not count: the use of numbers in qualitative research. Res Nurs Health. 2001;24(3):230–40. doi: 10.1002/nur.1025 11526621

